# First Report of Seroprevalence and Risk Factors of* Neospora caninum* Infection in Tibetan Sheep in China

**DOI:** 10.1155/2018/2098908

**Published:** 2018-05-29

**Authors:** Lan-Bi Nie, Wei Cong, Yang Zou, Dong-Hui Zhou, Qin-Li Liang, Wen-Bin Zheng, Jian-Gang Ma, Rui Du, Xing-Quan Zhu

**Affiliations:** ^1^State Key Laboratory of Veterinary Etiological Biology, Key Laboratory of Veterinary Parasitology of Gansu Province, Lanzhou Veterinary Research Institute, Chinese Academy of Agricultural Sciences, Lanzhou, Gansu Province 730046, China; ^2^College of Animal Science and Technology, Jilin Agricultural University, Changchun, Jilin Province 130118, China; ^3^College of Marine Science, Shandong University at Weihai, Weihai, Shandong Province 264209, China; ^4^Key Laboratory of Fujian-Taiwan Animal Pathogen Biology, College of Animal Sciences, Fujian Agriculture and Forestry University, Fuzhou, Fujian Province 350002, China; ^5^College of Veterinary Medicine, Northwest A&F University, Yangling, Shaanxi Province 712100, China; ^6^Jiangsu Co-Innovation Center for the Prevention and Control of Important Animal Infectious Diseases and Zoonoses, Yangzhou University College of Veterinary Medicine, Yangzhou, Jiangsu Province 225009, China

## Abstract

*Neospora caninum* is an intracellular protozoan parasite which can cause abortion and stillbirth in ruminants. However, there is no information on Tibetan sheep* N. caninum* infection in China. A total of 2187 serum samples were collected from Tibetan sheep in the major production areas of Luqu, Maqu, and Tianzhu in Gansu province, and Nyingchi in southeast Tibet, China. All samples were analyzed for the presence of antibodies to* N. caninum* using a competitive-inhibition enzyme-linked immunoassay. Of the 2187 serum samples, 184 (8.4%, 95% CI 7.3-9.6) were tested* N. caninum* seropositive. The* N. caninum* seroprevalence ranged from 4.4% (95% CI 1.4–7.4) to 11.3% (95% CI 8.2–14.4) among different regions, seasons, ages, and pregnancies, and there was no statistical significance among those groups (*P* > 0.05). Seroprevalence in male (10.8% 69/638) (95% CI 8.4–13.2) was significantly higher than in female (7.4% 115/1549) (OR =1.51, 95% CI 6.1–8.7) (*P* < 0.01). To our knowledge, this is the first report of* N. caninum* seroprevalence in Tibetan sheep in China, which provides baseline data for the prevention and control of* N. caninum* infection in Tibetan sheep.

## 1. Introduction

*Neospora caninum *is an obligate intracellular protozoan which can infect a variety of mammals and birds [1]. During the whole life cycle of* N. caninum*, canids are the exclusive definitive hosts and shed environmentally resistant oocysts [2]. Many ruminants like sheep, goats, and dairy cattle commonly act as the primary intermediate hosts and are subjected to abortion, stillbirths, and neonatal mortalities [3, 4]. Both vertical transmission from infected mother to foetus and horizontal transmission from food or water contaminated with oocysts can result in the infection of* N. caninum *[5]. Though no* N. caninum* has been found from human tissue till now, antibodies to* N. caninum* have been detected in human serum [6], suggesting a zoonotic potential of the parasite.

The* N. caninum* seroprevalence has been reported in sheep and goats worldwide [7–9]. In China's Tibetan areas, the prevalence and infection of* N. caninum* have been reported in black yaks [10, 11] and white yaks [12]. However, there is no information about the infection of* N. caninum* in Tibetan sheep in China. Tibetan sheep is one of China's three major varieties of sheep, mainly inhabiting in the Qinghai-Tibet Plateau [13]. Tibetan sheep produce high quality pelage, provide nutritive and delicious meat, and are economically important for the local Tibetans. Thus, the objective of this study was to determine the seroprevalence and assess rick factors of* N. caninum* infection in Tibetan sheep in China.

## 2. Materials and Methods

### 2.1. The Study Sites

In the present study, serum samples were collected from Tibetan sheep in Gansu province (32°31′′~42°57′′ N, 92°13′′~108°46′′ E), northwest China, and Nyingchi prefecture (26°52′′~30°40′′ N, 92°09′′~98°47′′ E) in southeast Tibet, southwest China. The average elevation of the surveyed areas is more than 3000 meters above sea level and has a plateau continental climate.

### 2.2. Serum Samples

Serum samples were collected from Tibetan sheep via the caudal vein by local veterinary practitioners. A total of 2187 serum samples were collected from farmed Tibetan sheep from Luqu, Maqu, and Tianzhu in Gansu province, and Nyingchi in Tibet, China (Table 1). Serum samples were taken to the laboratory and kept at room temperature for 2 hours and then centrifuged at 3000 rpm for 10 min. Serum was separated and stored at – 20°C until further tested. Moreover, a standardized questionnaire was used to record region, age, gender, history of pregnancy, and sampling season.

### 2.3. Serological Examination

All samples were analyzed for the presence of antibodies to* N. caninum* using a competitive-inhibition enzyme-linked immunosorbent assay (cELISA) kit (VMRD, Pullman, USA) validated for ruminants according to the manufacturer's instructions. The test results were expressed as percentage of inhibition (%*I*) according to the following formula: %*I* = 100 [1- (Sample OD ÷ NC OD)]. The serum was examined in duplicate and considered positive if more than 30% inhibition was detected.

### 2.4. Statistical Analysis

Variables associated with* N. caninum* infection among Tibetan sheep of different seasons, regions, pregnancy, gender, and age groups were analyzed in a multivariable logistic regression model, and probability (*P*) value < 0.05 was considered as statistically significant between factors and prevalence. Odds-ratios (OR) with 95% confidence intervals based on likelihood ratio statistics are calculated. All statistical analyses were performed using the SAS (Statistical analysis system, Version 8.0).

## 3. Results and Discussion

In this study, the total seroprevalence of* N. caninum* among the examined Tibetan sheep was 8.4% (184/2187, 95% CI 7.3-9.6). The* N. caninum* prevalence in Tibetan sheep varied between regions, ranging from 4.4% in Luqu (8/182, 95% CI 1.4-7.4) to 9.4% in Tianzhu (90/962, 95% CI 7.5-11.2) (Table 1), but the difference was not statistically significant (*P *> 0.05). Caprine infection of* N. caninum* has been reported worldwide, for example, the lower prevalence of 5.6% in Iraq [14], 6.1% in Costa Rica [15], 6% in the Czech Republic [16], 6.6% in Argentina, and 6.4% in Brazil [17], the higher prevalence of 25.9% in Turkey [18] and 23.6% in Thailand [19]. Due to the different investigation sites, different detection methods, sheep breeds, and various sample capacities, it is difficult to compare* N. caninum* prevalence between these studies, but these studies all confirmed that caprines are truly easy to be infected by* N. caninum*.

To evaluate the seasonal difference of* N. caninum *seroprevalence in Tibetan sheep, serum samples were collected in the four seasons during a whole year. The results indicated that* N. caninum *infection varied in a year ranging from 6.5% in autumn to 11.3% in summer, but the difference was not statistically significant (*P* > 0.05). High altitude in the Qinghai-Tibet Plateau results in significant temperature differences between day and night, which may, to some degree, weaken the influence of seasons on the* N. caninum *infection in Tibetan sheep.

Statistically, the* N. caninum* seroprevalence in male Tibetan sheep (10.8%) (69/638, 95% CI 8.4-13.2) was significantly higher than in the female (7.4%) (115/1549, 95% CI 6.1–8.7) (*P *< 0.01), which is probably related to the different hormone levels between males and females [20]. It also may be due to horizontal transmission of* N. caninum* and exposure of older Tibetan sheep for a long period. But this result is not consistent with some previous reports that no significant differences were observed between male and female goats [21–23], probably reflecting the breed difference.

In addition, the effects of age and pregnancies on the* N. caninum* infection were also evaluated among the examined Tibetan sheep, and the results indicated age and pregnancies had no significant effect on the* N. caninum* seroprevalence (both* P *> 0.05), although these results were not consistent with some previous studies when the older and pregnant animals were at high risk of being exposed to* N. caninum *[24].

In conclusion, this is the first report of the* N. caninum* seroprevalence and risk factors associated with the parasite in Tibetan sheep in China. It provides baseline data for establishing control programs. In the future, further molecular investigations and bioassays on* N. caninum* infection in Tibetan sheep should be carried out.

## Figures and Tables

**Table 1:** Seroprevalence of *Neospora caninum *in Tibetan sheep from different regions, genders, seasons, ages, and pregnancies in Gansu and Tibet provinces, China, tested by competitive-inhibition enzyme-linked immunosorbent assay.

Variable	Category	No. examined	Positive (%)	95% CI	*P*-value	OR (95% CI)
Region	Luqu	182	8 (4.4%)	1.4-7.4	>0.05	Reference
	Maqu	588	48 (8.2%)	5.9-10.4		1.9 (0.9-4.2)
	Tianzhu	962	90 (9.4%)	7.5-11.2		2.3 (1.1-4.7)
	Nyingchi	455	38 (8.4%)	5.8-10.9		1.9 (0.9-4.3)
Sex	Male	638	69 (10.8%)	8.4-13.2	<0.01	1.5 (1.1-2.1)
	Female	1549	115 (7.4%)	6.1-8.7		Reference
Season	Spring	480	41 (8.5%)	6.2-11.1	>0.05	1.4 (0.8-2.2)
	Summer	398	45 (11.3%)	8.2-14.4		1.8 (1.1-2.9)
	Autumn	479	31 (6.5%)	4.3-8.7		Reference
	Winter	375	29 (7.7%)	5.0-10.4		1.2 (0.7-2.1)
	No data	455	38 (8.4%)	5.8-10.9		1.3 (0.8-2.1)
Age (year^*∗*^)	0-1	447	45 (10.1%)	7.3-12.9	>0.05	1.9 (1.1-3.8)
	≥1-2	413	27 (6.5%)	4.2-8.9		1.2 (0.6-2.5)
	≥2-3	243	13 (5.4%)	2.5-8.2		Reference
	≥3	1084	99 (9.1%)	7.4-10.9		1.8 (0.9-3.2)
Pregnancy	Not pregnant	581	49 (8.4%)	6.2-10.7	>0.05	1.2 (0.8-1.7)
	Pregnant	796	59 (7.4%)	5.6-9.2		Reference
	No data	810	76 (9.4%)	7.4-11.4		1.3 (0.9-1.9)
Total		2187	184 (8.4%)	7.3-9.6		

95% CI, 95% confidence interval; OR, odds ratio.

^*∗*^Animals are grouped by age of year.

## Data Availability

The* Neospora caninum* prevalence data used to support the findings of this study are included within the article.

## References

[1] Basso W., Moré G., Quiroga M. A., Balducchi D., Schares G., Venturini M. C. (2014). *Neospora caninum* is a cause of perinatal mortality in axis deer (*Axis axis*). *Veterinary Parasitology*.

[2] Dubey J. P., Jenkins M. C., Kwok O. C. H. (2013). Congenital transmission of *Neospora caninum* in white-tailed deer (*Odocoileus virginianus*). *Veterinary Parasitology*.

[3] Dubey J. P., Leathers C. W., Lindsay D. S. (1989). *Neospora caninum*-like protozoon associated with fatal myelitis in newborn calves. *Journal of Parasitology*.

[4] Barr B. C., Bjerkås I., Buxton D. (1997). Neosporosis: report of the international *Neospora* workshop. *Compendium on Continuing Education for the Practicing Veterinarian*.

[5] Dubey J. P., Schares G., Ortega-Mora L. M. (2007). Epidemiology and control of neosporosis and *Neospora caninum*. *Clinical Microbiology Reviews*.

[6] Tranas J., Heinzen R. A., Weiss L. M., Mcallister M. M. (1999). Serological evidence of human infection with the protozoan *Neospora caninum*. *Clinical & Diagnostic Laboratory Immunology*.

[7] Dubey J. P., Hartley W. J., Lindsay D. S., Topper M. J. (1990). Fatal congenital *Neospora caninum* infection in a lamb. *Journal of Parasitology*.

[8] Kobayashi Y., Yamada M., Omata Y. (2001). Naturally-occurring *Neospora caninum* infection in an adult sheep and her twin fetuses. *Journal of Parasitology*.

[9] Eleni C., Crotti S., Manuali E. (2004). Detection of *Neospora caninum* in an aborted goat foetus. *Veterinary Parasitology*.

[10] Ma L., Shen Y. (2006). Serological diagnosis of *Neospora caninum* infection in yaks in Qinghai, China. *Chinese Medical Journal*.

[11] Liu J., Cai J. Z., Zhang W. (2008). Seroepidemiology of *Neospora caninum* and *Toxoplasma gondii* infection in yaks (*Bos grunniens*) in Qinghai, China. *Veterinary Parasitology*.

[12] Meng Q.-F., Yao G.-Z., Qin S.-Y. (2017). Seroprevalence of and risk factors for *Neospora caninum* infection in yaks (*Bos grunniens*) in China. *Veterinary Parasitology*.

[13] Li W. C. (2012). Detection of antibodies against *Toxoplasma gondii*, *Chlamydia abortus* and *Bacterium burgeri* in Tibetan sheep. *Chinese Journal of Veterinary Medicine*.

[14] Figliuolo L. P. C., Kasai N., Ragozo A. M. A. (2004). Prevalence of anti-*Toxoplasma gondii* and anti-*Neospora caninum* antibodies in ovine from São Paulo State, Brazil. *Veterinary Parasitology*.

[15] Moore D. P., de Yaniz M. G., Odeón A. C. (2007). Serological evidence of *Neospora caninum* infections in goats from La Rioja Province, Argentina. *Small Ruminant Research*.

[16] Bartova E., Sedlak K. (2012). *Toxoplasma gondii* and *Neospora caninum* antibodies in goats in the Czech Republic. *Veterinarni Medicina*.

[17] Ghattof H. H., Faraj A. A. (2015). Seroprevalence of *Neospora caninum* in goats in Wasit province, Iraq. *International Journal of Current Microbiology and Applied Science*.

[18] Dubey J. P., Lindsay D. S. (1996). A review of *Neospora caninum* and neosporosis. *Veterinary Parasitology*.

[19] Cayvaz M., Karatepe M. (2011). Seroprevalence of *Neospora caninum* in goats in Niğde province. *Kafkas Universitesi Veteriner Fakultesi Dergisi*.

[20] Gharekhani J., Heidari H. (2014). Serology based comprehensive study of *Neospora* infection in domestic animals in Hamedan province, Iran. *Journal of Advanced Veterinary and Animal Research*.

[21] García-Bocanegra I., Cabezón O., Pabón M. (2012). Prevalence of *Toxoplasma gondii* and *Neospora caninum* antibodies in Spanish ibex (*Capra pyrenaica hispanica*). *The Veterinary Journal*.

[22] Topazio J. P., Weber A., Camillo G. (2014). Seroprevalence and risk factors for *Neospora caninum* in goats in Santa Catarina state, Brazil. *Revista Brasileira de Parasitologia Veterinária*.

[23] Gharekhani J., Esmaeilnejad B., Rezaei H., Yakhchali M., Heidari H., Azhari M. (2016). Prevalence of anti-*Neospora caninum* antibodies in Iranian goats. *Annals of Parasitology*.

[24] Cerqueira-Cézar C. K., Pedersen K., Calero-Bernal R., Kwok O. C., Villena I., Dubey J. P. (2016). Seroprevalence of *Neospora caninum* in feral swine (*Sus scrofa*) in the United States. *Veterinary Parasitology*.

